# A glance of the blood stage transcriptome of a Southeast Asian *Plasmodium ovale* isolate

**DOI:** 10.1371/journal.pntd.0007850

**Published:** 2019-11-15

**Authors:** Awtum M. Brashear, Wanlapa Roobsoong, Faiza A. Siddiqui, Wang Nguitragool, Jetsumon Sattabongkot, Margarita M. López-Uribe, Jun Miao, Liwang Cui

**Affiliations:** 1 Department of Entomology, Pennsylvania State University, Department of Entomology, University Park, Pennsylvania, United States of America; 2 Department of Internal Medicine, University of South Florida, Tampa, Florida, United States of America; 3 Mahidol Vivax Research Unit, Faculty of Tropical Medicine, Mahidol University, Thailand; 4 Department of Molecular Tropical Medicine and Genetics, Faculty of Tropical Medicine, Mahidol University, Thailand; Temple University, UNITED STATES

## Abstract

*Plasmodium ovale* accounts for a disproportionate number of travel-related malaria cases. This parasite is understudied since there is a reliance on clinical samples. We collected a *P*. *ovale curtisi* parasite isolate from a clinical case in western Thailand and performed RNA-seq analysis on the blood stage transcriptomes. Using both *de novo* assembly and alignment-based methods, we detected the transcripts for 6628 out of 7280 annotated genes. For those lacking evidence of expression, the vast majority belonged to the PIR and STP1 gene families. We identified new splicing patterns for over 2500 genes, and mapped at least one untranslated region for over half of all annotated genes. Our analysis also detected a notable presence of anti-sense transcripts for over 10% of *P*. *ovale curtisi* genes. This transcriptomic analysis provides new insights into the blood-stage biology of this neglected parasite.

## Author summary

Ovale malaria can be caused by one of two *Plasmodium* parasites, *P*. *ovale curtisi* and *P*. *ovale wallikeri*. *P*. *ovale* parasites are especially adept at evading prophylactic antimalarial drugs and traveling internationally, which makes them interesting from a global health perspective. Due to the lack of a continuous culture system for these parasites, research on *P*. *ovale* parasites has lagged behind and relies on clinical samples. Recent genome sequencing of a few *P*. *ovale* clinical isolates provides the blueprint of the parasite genome and *in silico* prediction of parasite genes. However, confirmation of the annotated genes and proof of their expression are needed. Here we obtained a *P*. *ovale curtisi* clinical isolate from western Thailand and performed RNA-seq analysis on the blood-stage parasites. High-quality RNA-seq data has enabled us to identify transcripts for 6628 of the 7280 annotated genes. Consistent with the blood stage development, housekeeping genes such as those involved in translation and metabolism are highly expressed. Prediction of the UTRs as well as detection of anti-sense transcripts and potential splicing patterns suggests the presence of complex gene regulation mechanisms for this parasite. This transcriptome dataset will serve as a useful resource for future studies of *P*. *ovale*.

## Introduction

*Plasmodium ovale* parasites represent a hurdle for malaria elimination programs [1]. Prevalence of *P*. *ovale* infection varies geographically; in most areas *P*. *ovale* accounts for 5% or less of the total malaria burden [2,3], but in some regions this number has been shown to exceed 10% [4]. Burden due to *P*. *ovale* is likely underestimated due to underperformance of the diagnostic tests for this parasite [5], and frequent misdiagnosis as other species [1,6,7]. As a result, this parasite is often mistreated and patients frequently suffer from relapses [1,7]. Further, *P*. *ovale* is known to have particularly long latency periods which allows evasion of prophylaxis [1,7–9]. Consequently, a disproportionately large number of *P*. *ovale* infections are identified among international travelers [1,10]. In those who take prophylactics before and during travel, the number of imported *P*. *ovale* cases outnumbers any other species [11].

Despite the importance of *P*. *ovale*, our understanding of its fundamental biology is still rudimentary. Although historically there were regions with significant morbidity due to *P*. *ovale*, this parasite has been neglected partially because it is rarer and causes less severe disease than *P*. *falciparum* [3]. Additionally, the lack of an *in vitro* culture system for *P*. *ovale* has led to an absolute reliance on field samples, which often have relatively low parasitemias and a high frequency of mixed infections. To further muddle our understanding of *P*. *ovale* malaria, it was recently discovered that it is caused by two distinct subspecies, *Plasmodium ovale curtisi* and *Plasmodium ovale wallikeri* [3, 5, 12–14].

Analysis of *P*. *ovale* genomes has shed light on the biology of *P*. *ovale* [12,13]. It has been conjectured that differences of this parasite from other malaria parasites could play a role in the adaptation *P*. *ovale* to certain hosts, blood types or life cycle durations. Transcriptomic and proteomic analyses can greatly complement genomic data. In parasites, unique biology such as monoallelic expression of surface antigens (important for immune evasion) is often regulated at the transcription level. Transcriptional activity can be regulated through processes such as anti-sense transcription and alternative splicing [14,15]. Meanwhile, the 5’ or 3’ untranslated regions (UTRs) can impact post-transcriptional processing of the transcript [16,17]. Using these mechanisms, *Plasmodium* species show considerable variations in gene expression throughout their life cycle [18,19], which might reflect adaptations to a variety of hosts and environments. Understanding RNA expression within a parasite can, therefore, provide many clues to its biology. Here, we present the first transcriptional analysis of the blood stages from a *P*. *ovale curtisi* clinical sample. Specifically, we examined abundantly expressed genes, splicing patterns including UTRs, and anti-sense transcription.

## Methods

### Ethics statement

The human subjects protocol was approved by the Ethical Review Committee of Faculty of Tropical Medicine, Mahidol University (MUTM2011-040). Fresh isolates of *P*. *ovale* was collected from a patient with acute malaria attending the malaria clinic in Sangkhlaburi District, Kanchanaburi Province, western Thailand. Written informed consent was obtained prior to sample collection.

### Parasite culture and enrichment

After diagnosis and consenting, 20 mL of venous blood were drawn from the patient into a heparinized tube. Whole blood was centrifuged at 800 xg for 10 min to removed plasma. After being washed once with the RPMI 1640 incomplete medium (ICM), the packed red blood cells (RBCs) were resuspended to 50% hematocrit with ICM and passed through Plasmodipur filter (Europroxima, Netherlands) to remove human leukocytes. Parasites were cultured in McCoy’s 5A medium supplemented with 25% human AB serum and incubated at 37°C under hypoxic condition (5% O_2_, 5% CO_2_, and 90% N_2_) for 20 h and light microscopy was performed to count parasite stages. After culture, RBCs were washed once with ICM and resuspended to 20% hematocrit with ICM. The resuspended RBCs were layered on 45% isotonic Percoll and centrifuged at 1,200 xg for 20 min without brake. Parasites were collected at the interface and washed three times with ICM for 10 min at 500 xg. The parasite pellet was put in 1 mL RNAprotect Cell Reagent and kept at -20°C. One microliter of parasite pellet was kept at -20°C for further DNA extraction.

### Extraction of parasite RNA and DNA

Purified parasites in RNAprotect Cell Reagent were thawed on ice and centrifuged at 5,000 xg for 10 min to discard supernatant. Parasite RNA was extracted using RNeasy Mini Kit (Qiagen) according to the manufacturer’s protocol with modifications. The RNA was eluted with 100 μL RNase-free water and kept at -80°C. Parasite DNA was extracted using DNeasy Blood and Tissue kit (Qiagen) using manufacturer’s protocol. The DNA was eluted by 100 μL of elution buffer and kept at -80°C.

### Whole genome amplification

Whole genome amplification was performed using REPLI-g mini kit (Qiagen) according to manufacturer’s protocol and using the included primers. For each amplification reaction, 5 μL of DNA sample was mixed with 5 μL denaturation buffer and incubated with 10 μL of neutralization buffer. The denatured DNA was then mixed with amplification buffer consisting of 29 μL of REPLI-g Mini reaction buffer and 1 μL of REPLI-g Mini DNA polymerase. Whole genome amplification was performed on thermal cycler at 30°C for 16 h followed by inactivation of REPLI-g Mini DNA polymerase at 65°C for 3 min. The amplified DNA was kept at -80°C.

### Library preparation and sequencing

Two cDNA libraries were prepared from extracted RNA using the Illumina TruSeq Stranded mRNA kit (LT-LS protocol). Total RNA was used directly for library preparation (non-selected) or poly-A selected with oligo-dT magnetic beads. After RNA fragmentation, the first strands of cDNAs were synthesized via reverse transcriptase PCR (RT-PCR) from random hexamers. Actinomycin D was added to the mix to prevent spurious DNA synthesis and improve strand specificity. The second strand was created with DNA polymerase I and RNase H, using dUTP rather than dTTP. This created blunt, strand-specific cDNAs, which were adenylated once to prevent chimeras. Indexing adapters were ligated to the end of cDNA to hybridize them to the sequencing cells for PCR amplification.

A whole-genome library was also prepared from the same *P*. *ovale* isolate using the Illumina TruSeq DNA PCR-Free kit. Genomic DNA was fragmented using Covaris shearing, and overhangs were fixed using a 3’ to 5’ exonuclease to remove the 3’ overhangs, and 5’ to 3’ polymerase to complement the 5’ overhangs. Adenylation and ligation were done similarly as described for the RNA-sequencing libraries except that PCR amplification was not used. The Poly-A selected and unselected RNA-sequencing libraries, and the genomic DNA library were all sequenced on the MiSeq. For quality control, DNA and RNA sequences were analyzed using fastqc v0.11, which displayed adapter content, read length, and sequencing quality. Trimmomatic was used to remove adapter sequences and reads where the sequencing quality dropped below a Phred score of 20 [20].

### Genome sequence trimming, assembly, and comparison

For DNA sequences, trimming and removing human DNA were performed by alignment to hg38 with BWA MEM, and remaining parasite reads were then aligned to PocGH01 (v3.0) using BWA mem [21]. To reduce the chance of misalignment affecting quality assurance results, alignment data was filtered for parts of the exome which had one copy using PlasmoCNVScan [22]. Variant calling was performed on aligned and filtered data using GATK HaplotypeCaller v.4.0 [23]. To rule out that the field sample might consist of more than one parasite, variable regions were used to plot minor allele frequencies (MAFs), and the analysis was confirmed with EstMOI v. 1.0 [24]. Concurrently, parasite reads were assembled *de novo* using velvet assembler v1.2 (k = 31) [25], scaffolded with sspace [26] and gaps were filled with gapfiller [27]. Quality metrics and comparisons to the reference PocGH01, as well as the additional references POC1 and POC2 [12], was performed using quast v. 4.6 [28]. Assembly and alignment data were both used to visually inspect individual hyper-variable genes, including merozoite surface protein 1 (*MSP1*) and circumsporozoite protein (*CSP*), for signs of sequence variation using Integrative Genome Viewer v.2.3 [29]. Additional PCR and Sanger sequencing were performed on CSP to confirm a repetitive insertion. To determine if the *P*. *ovale* sample belonged to *P*. *ovale wallikeri* or *P*. *ovale curtisi*, the assembled *MSP1* gene was extracted via Blast and compared to *MSP1* sequences from both *P*. *ovale* subspecies. For confirmation, we also downloaded sequences of PocRBP2 and PowRBP2 [30] and extracted the assembled RBP2 gene via sequence alignment.

### RNA-seq analysis by alignment to an existing genome and *de novo* assembly

Quality-controlled RNA-seq reads were aligned to the human genome hg38 using HiSat2 v1.6 [31] to remove contaminant human DNA. Reads not matching human sequences were aligned to the PocGH01 genome sequence (PlasmoDB release 34) using the RF parameter for strandedness. We used featureCounts to extract the corresponding number of reads mapping to each of the 7280 annotated *P*. *ovale curtisi* genes. To account for length bias, reads per kilobase (RPK) was calculated by dividing the number of reads over the length of the gene in kilobases, and the total of all RPKs divided by 1x10^6^ was accepted as the normalization factor. Transcripts per million (TPM) was calculated by dividing each gene’s RPK by the normalization factor. Genes with a TPM of 1 or higher were considered expressed in additional analyses.

Goatools provides enrichment and depletion values based on the available gene annotations from PlasmoDB [32]. Given that the majority of genes do not have any GO annotation, we also used the Word Enrichment function on PlasmoDB to obtain a more complete picture of enriched gene categories. Since PlasmoDB only offers enrichment of terms as opposed to goatools which also tests for signs of depletion, we performed the same analysis on the 623 genes which did not meet the threshold for transcription to find “depleted” terms. Gene ontology terms or phrases which were significantly enriched or depleted at a Bonferroni-adjusted p-value of 0.05 were accepted. Finally, we quantified relative expression of functional groups in our data. We used custom scripting to extract the orthologous Malaria Parasite Metabolic Pathways (MPMP) [33] for each of 7280 genes, as well as all annotated Gene Ontology groups. Each functional grouping was characterized by TPM, which was compared to the overall gene TPM distribution using a Wilcoxon rank sum test. Final results were adjusted for multiple testing with Bonferroni correction. All plotting was done with ggplot2 [34].

Since genome annotation for *P*. *ovale curtisi* is incomplete, and alignment-based methods can omit areas of poor genome annotation, we further analyzed the presence of multi-gene families by *de novo* assembly using Trinity v2.8 [35]. We processed the resulting assembled transcripts by filtering out those with TPM of less than one, then condensing similar transcripts at a 0.97 identity threshold using cd-hit-est v.4.7 [36]. We compared both steps individually as well as combined using an RSEM-EVAL score [37] and the TrinityStats.pl companion script for trinity. RSEM-EVAL scores were very similar across all four categories (unprocessed, selected for TPM, condensed, and selected for TPM then condensed), so we chose the assembly which had the fewest unsupported contigs and the largest N50.

### Function prediction and gene families

Transcript assemblies were compared to annotated predicted *P*.*ovale curtisi* proteins from PlasmodDB using Blastx in order to establish dataset overlap. Because we aimed to establish gene families by use of domain structure, we predicted peptides with Transdecoder v. 5.0. Predicted peptides were compared to the Uniprot database using blastp, and these results were compared to blastx results. After peptide predictions were accepted, we extracted all sequences belonging to five protein families (PIR, STP1, tryptophan-rich antigens, PHIST and ETRAMP) from all five human *Plasmodium* species annotated on PlasmoDB. Similar proteins were condensed using cd-hit (identity threshold 0.90) in order to simplify the data set, aligned using Muscle v 3.8 and used to construct a protein profile using hmmbuild from HMMer v.3.1 [38]. Predicted peptides with an e value of 1E-5 or better to any of the annotated family peptides after BlastP were compared to the peptide profile with hmmscan. Peptides matching the profile with an e-value of 1E-5 or lower, at least as large as the smallest peptide in the database, were accepted as family members.

### Comparison to gene expression profiles of *P*. *vivax*

To understand the scope of our data with respect to the parasite life cycle, we compared our sample to existing time-series RNA-seq data. Reference-aligned reads were compared to existing time course data for *P*. *vivax* [19]. Data was downloaded from the NCBI Sequence Read Archive, processed similarly as above (except that we neglected strandedness, used the Sal-I *P*. *vivax* reference, and used paired-end sequencing when necessary) and count data was extracted using featureCounts v1.6.0 [39]. Genes were converted to ortholog groups according to PlasmoDB. Each time point was averaged over the two study samples (with the exception of the 12-h time point and our sample for which only one sample existed, each). Log_2_FoldChange was then calculated compared to the mixed time-point data. Presented orthologs were those deemed to have conserved expression profiles [18]. Spearman’s correlation analysis was performed against the *P*. *ovale* field sample for each *P*. *vivax* sample and DeSeq2 was used to perform differential expression against ortholog groups published for *P*. *vivax* [40].

### Isoform structure and UTR predictions

To identify alternative splicing, Cufflinks [41] was used to extract isoform and gene-level feature data from the poly-A selected RNA-seq data. Potential novel isoforms output by cufflinks and cuffcompare were individually inspected in the Integrated Genome Viewer, and examples of change in exonic variation were chosen for illustration.

To predict UTRs on both sides of the open reading frames (ORFs), we used Transdecoder peptide prediction. We extracted the locations of any 5’ or 3’ UTR predicted on a partial or complete ORF and subtracted its end point from its start point (relative to the coding gene) to establish its length—in the case of 3’ UTR this resulted in a negative number. We plotted all of these as a general guide to UTR length. The presence of multiple isoforms can result in differing UTR lengths. Thus, to be generalizable during annotation of individual genes, all predicted coding sequences of a respective gene were aligned to that gene, and the longest UTR length was accepted.

### Identification of anti-sense transcripts

To understand the prevalence of anti-sense transcripts within *P*. *ovale*, reads without poly-A selection were quality-controlled and aligned to the genome as explained above. For each gene in the annotated *P*. *ovale* genome, featureCounts v.1.6 was used to gather counts in both the sense and anti-sense directions from the reference-aligned reads. For any gene that had over 5 total read counts we calculated the proportion of all reads which were in the antisense direction. The proportion was divided into 0, >0–0.25, >0.25–0.5, >0.5–0.75 and >0.75–1 and these groups were used as proxies for different degrees of anti-sense regulation. Genes falling in each group were input into PlasmoDB for word enrichment analysis, where terms with a Bonferroni-adjusted P-value of 0.05 or less were used as representative. As an exception, the third group did not display any word enrichment trends, so instead we used gene ontology enrichment characterization across biological processes, cellular components and molecular functions, which did not produce additional results in any other group.

## Results

### Genomic analysis of *P*. *ovale curtisi*

We obtained a clinical isolate of *P*. *ovale* from western Thailand and performed genome and transcriptome analysis on this parasite. After whole genome amplification and sequencing, we obtained 17,518,533 paired-end reads of 250 bp. After removing human reads and poor-quality sequences, 13,048,280 reads were kept and aligned to the reference PocGH01, which was assembled from three *P*. *ovale curtisi* and *P*. *falciparum* co-infections acquired in Ghana and Cameroon [13]. The average depth of coverage per base was 150x; however due to whole genome amplification, coverage was unequally distributed with 1,496,169 bp having a depth of 10 reads or less and 1,082,107 having no coverage at all. Initial call identified 3,525,768 single nucleotide polymorphisms (SNPs) across the genome. After filtering for low-quality or intergenic SNPs and those mapping to genes estimated to have more or less than 1 copy, 120,009 SNPs were retained for analysis. *De novo* assembly resulted in 35,915 contigs. After removing small contigs, 6918 scaffolds totaling 27.3 MBP remained, of which 26.6 MBP aligned to PocGH01, representing 79% of the genome. Because there is no finished assembly and we wanted to ensure we were using an optimal reference genome, we also aligned the assembled genome to alternative *P*. *ovale curtisi* assemblies POC1 and POC2 [12], and found that 26.2 and 26.3 MBP of the assembled genome aligned to them, respectively. Out of 7280 genes annotated for PocGH01, we assembled 4530 genes and had partial representation of another 1963.

We performed a series of analyses to establish multiplicity of infection as well as subspecies identification of the parasite isolate. For each SNP, we extracted the proportion of reads belonging to the lesser of two variants, also known as the MAF. MAFs for individual SNPs were predominantly below 5% secondary variants (S1A Fig), which suggests a monoclonal infection. We visually validated that individual regions, such as *MSP1* and *CSP*, were monomorphic in nature using alignment data (S1B Fig). We extracted a complete *MSP1* gene sequence from the *de novo* assembled contigs, and compared it by pairwise alignment to previously published *MSP1* genes from five *P*. *ovale curtisi* and five *P*. *ovale wallikeri* isolates [42]. The *P*. *ovale* field sample shared 100% identity with 3 of the *P*. *ovale curtisi* (POC) samples while the lowest identity to any POC sequence was 99.69% (S1C Fig, S1 Table). Conversely, each of the *P*. *ovale wallikeri* (POW) sequences aligned to our sample in a minimum of two fragments, ranging between 90.21% and 94.12% identity. Further, we compared an extracted sequence of RBP2 to publicly RBP2 sequences from *P*. *ovale curtisi* and *P*. *ovale wallikeri* and found that the RBP2 sequence from our sample shared 100% identity with the PocRBP2, but only 97.35% identity with the PowRBP2.

To demonstrate the relevance of genomic variation between the western Thailand parasite and that in the PocPH01 reference, we also predicted functional outcomes of the 120,009 high-quality variants. Based on annotated coding sequences, we projected that 1253 genes have a variant with at least a moderate predicted impact on the coding sequence, with 91 genes conferring a high effect (S1 File). Of these 91, there are only 4 genes which are not either part of an exported, highly variable gene family (*PIR*, *STP1*, *RBP*) or an unannotated protein. They encode the predicted autophagy-related protein 23, *TSR2* pre-rRNA-processing protein, guanylyl cyclase beta, and DNA polymerase theta.

### Blood stage transcriptomic analysis

RNA-seq analysis of poly-A selected and non-selected libraries resulted in 12,917,696 and 12,808,819 150 bp single-end reads, respectively (Table 1). After trimming and quality control, 11,381,290 reads (88.1%) from the Poly-A selected data, while 9,976,224 reads (77.9%) from the non-selected data were aligned to the PocGH01 genome. In total, all processing resulted in average coverages of 57 x and 10 x, respectively. The majority of reads from both datasets aligned to annotated genes; 8,377,744 (64.9%) reads from the Poly-A selected library and 8,805,493 (68.7%) reads from the non-selected library mapped to the annotated genes. We additionally checked alignment to 83 genes corresponding to tRNA and rRNA, finding that while 143,694 (1.1%) reads from the Poly-A selected library aligned to tRNA or rRNA, 7,753,155 (60.5%) reads from the non-selected library mapped to these 83 tRNA and rRNA genes.

**Table 1:** Sequencing performance. *For assembly data, the genome and transcriptome had different assembly and post-assembly methodology. For these metrics report, we used all contigs.

	Genome	Poly-A Selected	Not Selected
**Read Length**	2x250bp	150bp	150bp
**Reads**	17,518,533	12,917,696	12,808,819
**Mapped to GH01**	13,048,280 (74.5%)	11,381,290 (88.1%)	9,976,224 (77.9%)
**Average Depth of Coverage**	150x	57x	10x
# Assembled Contigs*	25,122	35,916	NA
Assembly N50*	6,046	1,178	
**RNA reads aligned to annotated genes**	NA	8,377,744 (64.9%)	8,805,493 (68.7%)

**Fig 1:**
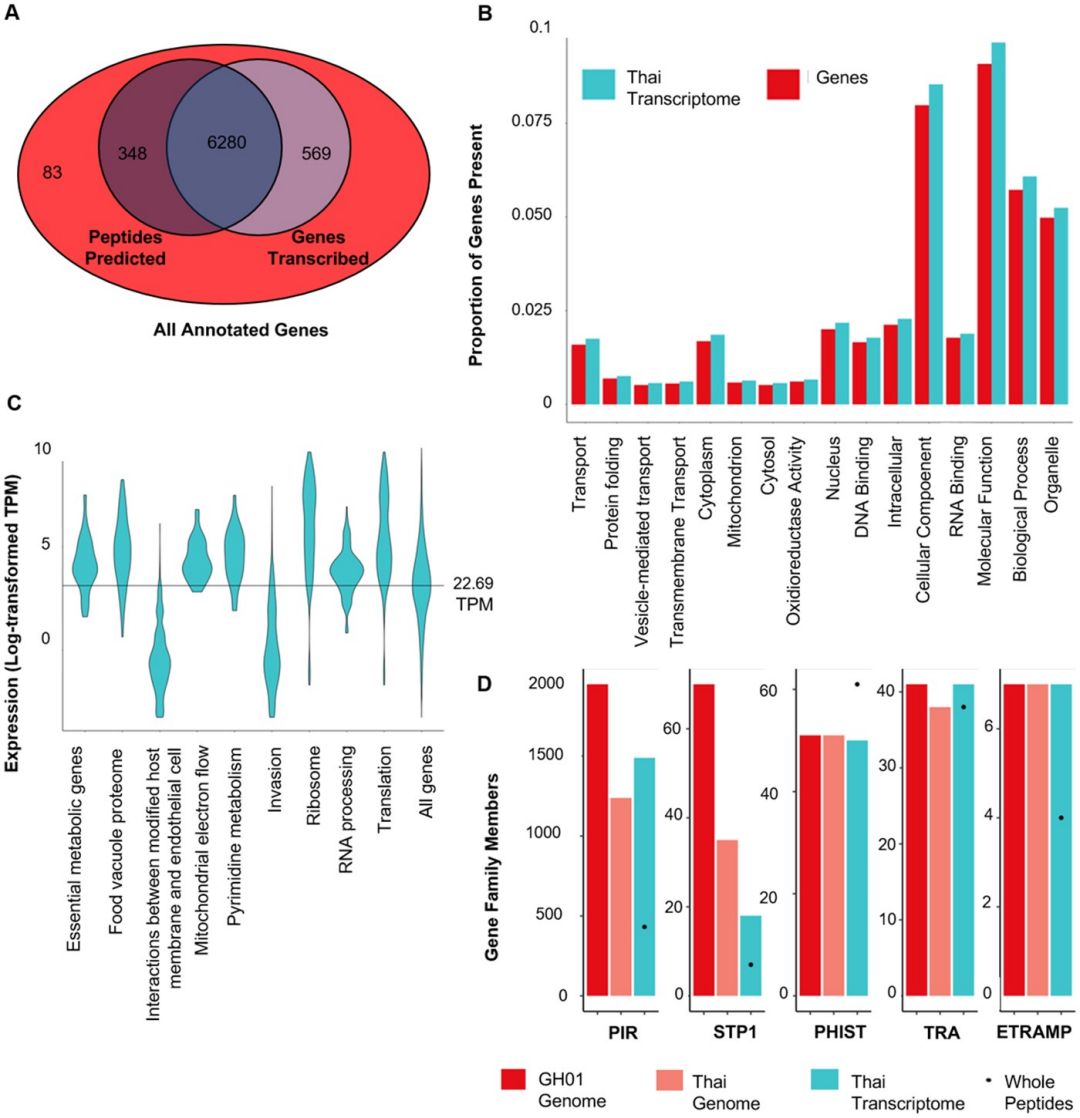
Gene expression within the *P*. *ovale curtisi* field sample. (A) Overlap between assembly and alignment-based methodology. Genes identified by peptide prediction on transcripts and transcript fragments are outlined in dark blue while the genes identifiable by transcription at TPM greater than or equal to 1 are in lighter blue. The red outer circle displays all annotated genes, including 83 of which were not identified by either means. (B) Proportion of genes annotated from the existing genome (PocGH01, PlasmoDB.org) compared to those found by alignment at 1 or more TPM in each of the statistically significant Gene Ontology groupings (Bonferroni-adjusted P-value < 0.05). (C) Distribution of TPM (Log transformed) per gene grouping with significantly high or low transcription in the *P*. *ovale* field sample. The line through the center represents the median of all genes. (D) Gene family repertoires. Bars represent genes above TPM 1, while points within the study bar represent whole peptides, defined as those with both a beginning and end present in the ORF.

These data provide transcription-level evidence for 6628 annotated genes (Fig 1A). Only 16 GO terms differed significantly from the accepted annotation in terms of the number of genes present (Fig 1B). All existing GO biases were enrichments compared to the accepted annotation, and the most drastic among these were the broad categories “Molecular Function”, “Biological Process” and “Cellular Component”. No GO term sets were found to be depleted. Of 652 genes for which transcripts were not found, 150 and 137 had complete and partial genome coverage, respectively, whereas 365 had no genome coverage.

Of the 652 genes without sufficient transcriptional evidence, 512 were *STP1* or *PIR* family members (S2 File). There were also 32 hypothetical proteins and 20 conserved *Plasmodium* proteins with unknown functions which were not significantly transcribed. The majority of the remaining unfounded genes were ribosomal proteins (18), snoRNA (11) and tRNA (25). There were just 34 unfounded genes which did not fall into any of these larger groups. We identified the *P*. *falciparum* orthologs of these genes and examined the expression profile in mosquito stages (oocysts and sporozoites) compared to asexual parasites [43]. We found that at least 7 genes not expressed in our data set have orthologs for which expression is substantially lower in blood stage parasites. These genes include a putative circumsporozoite protein (PocGH01_00239700), 6-cysteine protein P52 (PocGH01_03011900), two cysteine repeat modular proteins (PocGH01_03022100 and PocGH01_12078100), perforin-like protein 4 (PocGH01_05021400), secreted ookinete protein (PocGH01_11012800), and porphobilnogen synthase (PocGH01_12074600). Additionally, the ortholog of one gene (PocGH01_07030700, perforin-like protein 3) was reported as having notable asexual expression, but was also implicated in midgut invasion [44]. Additional genes which were not expressed in this sample include 3 kelt proteins, 3 RNA polymerase subunits, 2 copies of merozoite surface protein 3, and 2 AP2 transcription factors.

### Stage-specific expression patterns

Light microscopy of parasites revealed that our sample consisted of 81% trophozoites (590/728), 9.3% schizonts (68/728) and 9.6% gametocytes (70/728). To determine the degree to which our RNA sequencing analysis was biased by the predominance of life stages, we extracted 160 single-gene orthologs which have previously been shown to have conserved time-dependent transcription between 6 different *Plasmodium* species [18] and compared expression of these within our sample to *P*. *vivax* orthologs from each of 7 asexual blood stage time points [19]. The *P*. *ovale curtisi* sample formed a clade with expression profiles from *P*. *vivax* at 6, 12, 18 and 24 h post infection, and had the highest correlation with the 12 and 18 h post-infection samples (Spearman’s rho = 0.44), followed closely by the sample from 24 h (rho = 0.41) (S2 Fig). Notably, transcripts for the ookinete surface proteins P28 and P25 were high at 541.0 and 388.4 TPM, respectively. In other *Plasmodium* species, these genes are only expressed during gametocyte–ookinete development, confirming that the *P*. *ovale curtisi* blood-stage parasites contained gametocytes. Additionally, transcripts for P28 and a putative gamete release protein (PocGH01_01023000, TPM = 452.66) ranked within the top 5% of all genes. Further, we searched the *P*. *ovale curtisi* transcriptome for genes that are specific to sexual stages and identified a list of 29 genes indicative of the presence of gametocytes (S2 File). The median transcript abundance for this group was 52.14 TPM, which is significantly higher than the median for all *P*. *ovale* genes, 22.7 (P < 2.2x10^-16^, Wilcoxon rank sum). Finally, we extracted 14 liver and sporozoite stage genes which had a median TPM of 5.69, though this was not a statistically significant reduction (P > 0.05). Circumsporozoite protein (PocGH01_00239700) and liver specific protein 2 (PocGH01_03012800) were not expressed above the threshold.

### Abundantly expressed genes

We found 262 different functional clusters (168 GO terms and 88 metabolic pathway maps) for which the transcript abundance was significantly higher than that of the overall gene set (S3 File). Abundantly expressed gene groups included many pathways relevant to cellular metabolism such as “essential metabolic genes”, “food vacuole proteome”, “pyrimidine metabolism” and “mitochondrial electron flow” (Fig 1C, S2 File). There were also many functional groups with abundant expression pertaining to translational machinery such as “translation”, “ribosome” and “RNA processing” (Fig 1C, S2 File).

We further analyzed individual genes that were within the top 5% of abundance. The cutoff for the 95^th^ percentile was 422 TPM. Abundant genes were enriched for 15 different GO terms, including a notable number of terms relevant to translational activity including “translation”, “RNA binding”, “ribosome” and “structural constituent of the ribosome” (S3 File). In addition, there were 47 GO terms for which the term’s median expression level was significantly higher than the overall median expression level (Fig 1C, S3 File). Again, highly expressed terms included those pertaining to translational activity such as “translation”, “mRNA processing”, “RNA binding”, “ribosome”, “ribosome biogenesis” and “structural constituent of the ribosome”.

### Exported membrane proteins and invasion-related genes

There were 6 metabolic pathway maps in which gene expression was significantly lower than that of the total gene set. Among these were terms related to erythrocyte export such as “properties of proteins exported to erythrocyte”, “exported proteins with known location” and “interactions between modified host cell membrane and endothelial cell” (Fig 1B and 1C). Further analysis revealed that these groups contained many of the *PIR* and *STP1* gene family members, which had a TPM of less than 1. Another interesting low-expression group was “subcellular localization of proteins involved in invasion”, a group that did include *STP1* genes, but contained all 13 *P*. *ovale reticulocyte-binding protein* (RBP) genes. These *RBP* genes and pseudogenes were significantly under-represented with a median TPM of 7.05 as compared to overall gene expression (P = 0.007, Wilcoxon rank sum). Further examination of this group identified that only 4 genes of the 11 (3 copies of *RBP2b* and 1 copy of *RBP1a)* had a TPM over 10 (S2 File). Interestingly, despite some *RBP* genes having low expression, all 11, plus 4 pseudogenes, had a TPM over 1 and are therefore classified as transcriptionally active. One exception was that tryptophan-rich antigens were found to be abundantly expressed (median = 103.2 TPM, P = 2.6e-7, Wilcoxon rank sum).

### *STP1* and *PIR* genes

Of the 652 genes that lacked transcription in our dataset, 512 belong to either the *STP1* or *PIR* gene family (S2 File), which prompted us to perform further examination of multigene families. For the *STP1* and *PIR* gene families, 18 of the 70 predicted *STP1* genes and 1489 of the 1949 predicted *PIR* genes were detected at a TPM of ≥1 (Fig 1D). In contrast, for the *ETRAMP*, *PHIST* and *tryptophan-rich* gene families, transcripts were detected for 7 of 7, 50 of 51 and 41 of 41 genes, respectively, constituting complete or nearly complete representation of expected gene repertoires (Fig 1D). It is noteworthy that many *STP1* and *PIR* genes were also missing from the genome assembly. Of the combined partial and complete genes, 1238 belong to the *PIR* family, while 35 belong to the *STP1* family. Comparatively, we had at least partial representation of all 7 *ETRAMP* genes, 51 *PHIST* genes and 38 *tryptophan-rich antigen* genes (Fig 1D). Of the 366 genes not present in either the genome or transcriptome data, 317 are *PIR* genes, 28 are *STP1* genes and 1 is a *tryptophan-rich antigen* (S2 File).

Because alignment-based data can result in inclusion of partial transcripts, and because one transcript can code for many proteins, we performed *de novo* transcript assembly and peptide prediction. Our predicted peptides very closely matched alignment-based data with only 348 of 6628 genes being identified by read coverage that were not identified in the assembled transcripts (Fig 1A). However, for each of the five families the number of complete ORFs identified by assembly was lower than the number found via alignment alone (Fig 1D). One exception is the *PHIST* gene family for which a larger repertoire was found via assembly and peptide prediction than those annotated in the genome (Fig 1D).

### Different splicing patterns of transcripts

**Fig 2:**
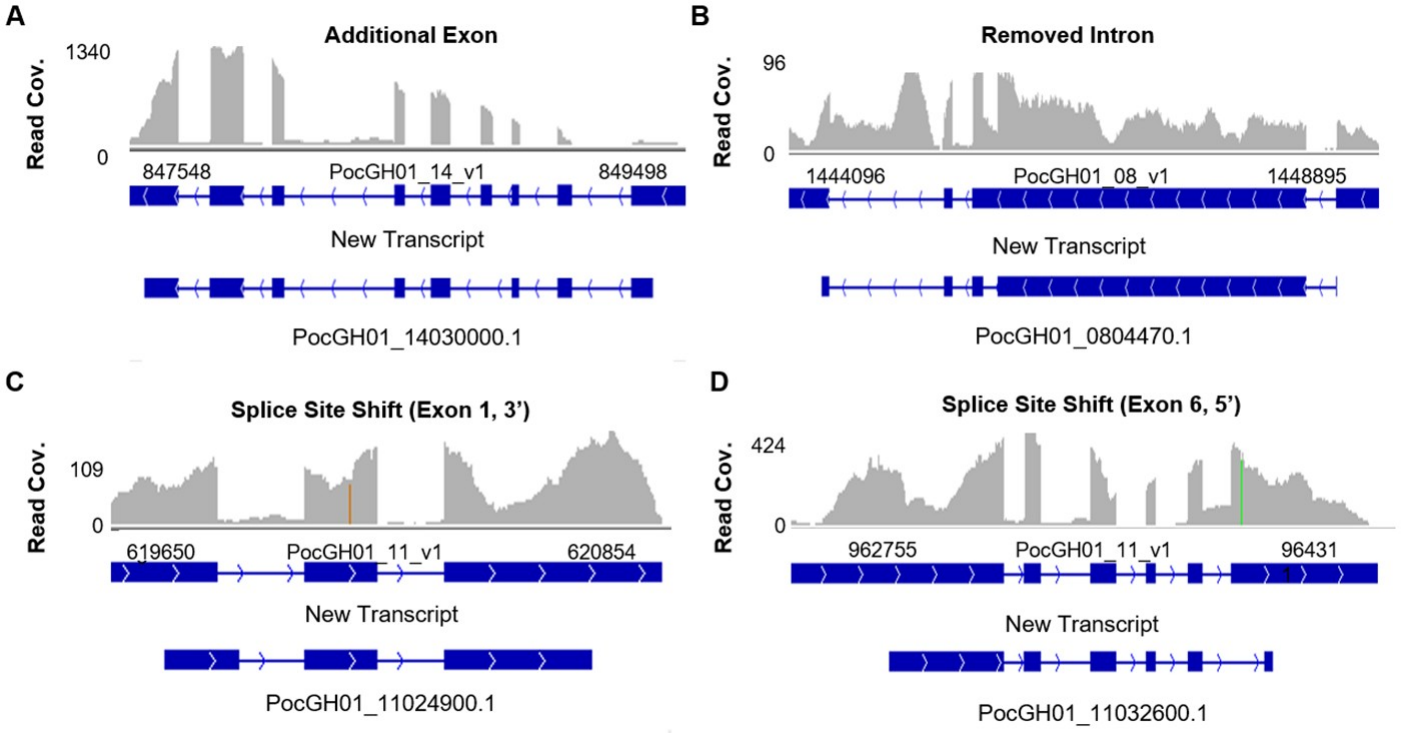
Examples of different splicing patterns in *P*. *ovale* clinical isolate. (A) New transcript identified with additional exon toward the center of PocGH01_14030000. (B) A missing intron in some transcripts of PocGH01_0804470. (C) A shift in splicing point resulting in a shorter intron for PocGH01_11024900. (D) A shift in splicing point resulting in a longer exon in PocGH01_11032600. For each the first panel represents read density, the second represents transcript structure.

We compared transcript structure to current annotations using a splice-aware aligner to predict alternative splicing and confirm the existing gene models. We found 2692 potential cases, corresponding to 1994 genes, wherein a transcript model was not identical to the current gene annotation (S2 File). In some cases, a transcript fragment was added or removed entirely (e.g., the 6^th^ exon was added in the new transcript for PocGH01_14030000) (Fig 2A). In others, a new splicing form was detected in addition to the previously annotated transcript (e.g., the removal of an intron in PocGH01_0804470) (Fig 2B). Deviation was often on a smaller scale such as slight shifts in the 3’ boundary of the 1^st^ exon in PocGH01_11024900 (Fig 2C) or the 5’ boundary of the last exon in PocGH01_11032600 (Fig 2D). In these examples and in most novel transcripts, either 5’, or 3’ UTRs or both were present (Fig 2).

### Prediction of UTRs

**Fig 3:**
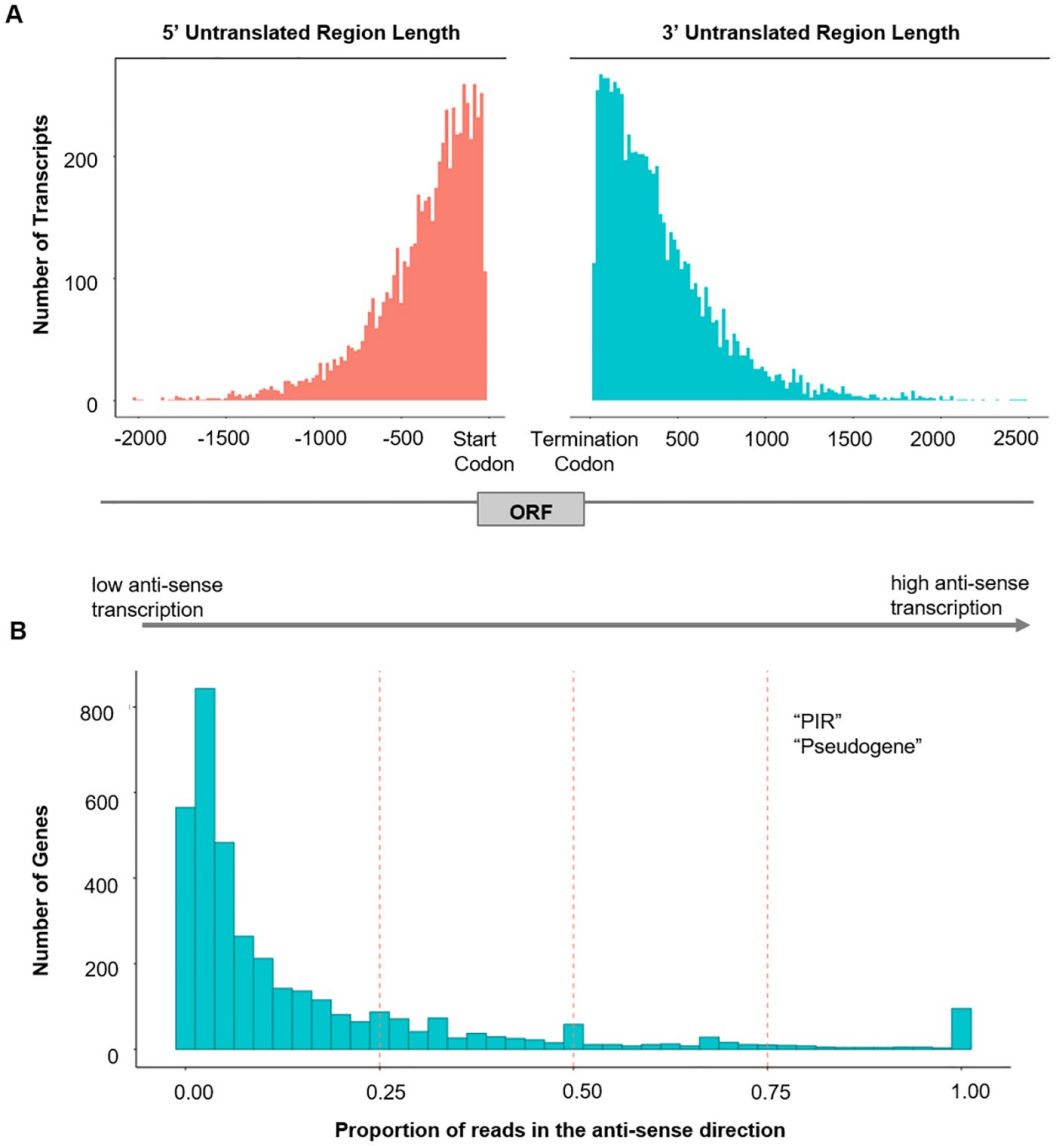
Trends in UTR length and anti-sense transcripts. (A) A histogram depicting the number of UTRs reaching each length (in bp) from all ORF predictions of assembled transcripts. To denote distance from coding section of the gene, five prime UTRs (left) were given negative values while three prime UTRs (right) were given positive values. The gene model on the bottom is depicted merely for visualization. (B) Distribution of proportion anti-sense reads by gene. Dotted lines separate genes into ranges and terms for each group represent terms significantly enriched (P > 0.05, Bonferroni-adjusted) within genes of that group.

We predicted ORFs from the assembled transcripts in order to infer UTRs (S3 File). For all possible transcript isoforms, 8442 possible 5’ UTRs were predicted with a median length of 290 bp and a maximum length of 2676 bp (Fig 3A); 10,342 possible 3’ UTRs were predicted with a median length of 311 and a maximum length of 3182 bp (Fig 3A). We mapped these isoforms back to 4570 annotated genes. Of these genes, the transcripts of 2273 genes contained both 5’ and 3’ UTRs. The longest 3’ UTR was found in PocGH01_00188801, a *PIR* gene, while the longest 5’ UTR was found in PocGH01_0601260, a gene annotated with domains corresponding to a calcium-transporting transmembrane protein.

### Detection of anti-sense transcripts

Using a strand-specific, non-selected, library we were able to detect 3657 genes with the presence of anti-sense transcripts. We divided genes into groups based on the proportion of anti-sense transcripts and performed word and GO enrichments (Fig 3B). The overall correlation between the proportion of anti-sense reads in the non-selected library, and transcript abundance within the Poly-A selected library was -0.04. However, among those genes with any anti-sense transcripts, the correlation was -0.38 (S3 Fig). For 2915 genes, anti-sense reads accounted for < 25% of transcripts. Within this low anti-sense group, transcripts were significantly more abundant than those with no anti-sense transcript (Wilcoxon Rank Sum, P < 2.2x10^-16^). For 742 genes, anti-sense reads accounted for more than 25% of total reads to the respective genes, and among these the level of transcripts was significantly lower than those with no anti-sense transcripts, and the reductions was most dramatic for those with the highest level of anti-sense transcripts (S3 Fig). Among the 152 genes with 75% or more anti-sense reads, the median non-selected TPM was 7.38, significantly less than reads with no anti-sense transcript (median TPM 28.26) and the total population (Wilcoxon Rank Sum, P < 2.2x10^-16^). Interestingly, *PIR* genes were enriched at this level, and were also enriched in genes with no anti-sense transcription, demonstrating a binary or “all-or-nothing” pattern of anti-sense transcription.

## Discussion

We have made the first transcriptome analysis of the blood stages of the neglected malaria parasite *P*. *ovale curtisi* using a clinical sample from Thailand. This sample represents blood stage parasites, with the presence of gametocytes. Transcripts were detected for 6628 genes which were previously annotated in the reference genome PocGH01. From the annotated genes, 366 genes were missing from our transcriptomic and genomic datasets. For over 2000 genes, we have found that existing models might be inaccurate or incomplete. Using *de novo* assembled transcripts and ORF predictions, we identified that over 4000 genes contained at least one UTR, with the median 5’ and 3’ UTR both being around 300 bp. Finally, using data derived from an additional library which was not poly-A selected, we were able to identify 742 genes wherein over 25% of transcripts was in the anti-sense direction.

*Plasmodium* parasites are known for extensive interspecies variation in terms of genetic content [12,13,45], gene expression [18,46], morphology [47], and life cycle components such as latency phase [11]. Study of *P*. *ovale* has been hindered by the lack of a sustainable *in vitro* culture system, the inability to readily distinguish it from other species, especially between the two *P*. *ovale* subspecies [7,48], the frequency of mixed infections [3], and low parasitemia [49]. Here, we provide the first glimpse into the transcriptome of this parasite, providing evidence of expression for 6628 out of the 7280 annotated genes. We found minimal bias in gene ontology of the genes found compared to the genes predicted from the published genome, suggesting our data is largely representative (Fig 1B). Of the genes which were not expressed, the majority (512 of 652) belonged to the *PIR* and *STP1* gene families, suggesting that not all of these genes are expressed. Another 54 belong to tRNA and snoRNA, while 55 belong to hypothetical proteins and proteins with unknown functions which could be transcriptionally inert or missing altogether; around 1/3 of these were not even partially present within the genomic data. The 34 unidentified proteins which are not attributable to any of these groups are likely to be transcriptionally inactive in this sample, and are diverse in predicted functions including polymerase subunits, transcription factors and surface proteins. Since only one of these genes, a tryptophan-rich antigen, is missing within the genomic data, these could be genes with conditional expression or which are transcriptionally inert. Accordingly, at least 8 genes which were not expressed were found to be orthologs of genes that are involved in mosquito stages within *P*. *falciparum* [43], while others including the liver specific protein 2 are likely specific to hepatic stages. It is noteworthy, however, that the majority of genes show at least some expression within blood stage parasites and very few seem to be specific to other stages. Since transcriptional activity varies within different *Plasmodium* species [46], unexpressed genes warrant further study within *P*. *ovale* to highlight the potential cause of their absence.

Consistent with their essential functions during parasite development, housekeeping genes were found to be abundantly expressed during blood stage development in *P*. *ovale*. For instance, genes involved in translation and essential metabolic processes were significantly enriched compared to other gene categories (Fig 1D, S3 File). This is supported by previous RNA quantification studies in multiple *Plasmodium* species, which have shown translational machinery genes to be among the most highly expressed regardless of parasite stages [19,50], indicating a high demand for translation and metabolism at all points in the parasite life cycle.

Gene expression in *Plasmodium* follows tightly regulated program with different sets of genes expressed in different stages [18,46], and this principle should also apply to *P*. *ovale*. The clinical *P*. *ovale* isolate was about 80% trophozoites with less than 10% schizonts and 10% gametocytes. Comparison of the gene expression pattern with that in *P*. *vivax* suggests that this Thai *P*. *ovale* isolate did not have a particularly strong correlation to any one stage, though it was mostly highly correlated to *P*. *vivax* parasites at 12, 18, and 24 h (S2 Fig). A high correlation to earlier stages within *P*. *vivax* agreed with the low abundance of invasion-related genes such as the RBP family members, which are normally expressed in schizont stage. However, it disagreed with our microscopy data which had schizonts but not rings, suggesting there may have been undetected young ring stages present as well. This could have been an outcome of short-term parasite culture. Of the 13 RBP family members detected, only 3 (2 copies of RBP2b and one copy of RBP1a) were detected with transcript abundance higher than 20 TPM. In addition, we also found relatively high abundance of certain sexual-stage genes such as *P25* and *P28*. In other *Plasmodium* species, P25 and P28 are major ookinete surface proteins transcribed in late-stage gametocytes and stored in RNA-protein granules as translationally repressed mRNAs [51,52]. Our data suggest the presence of potentially infectious gametocytes in the clinical samples, which corroborates the recent finding of readily-transmissible gameotcytes within *P*. *ovale* infected blood samples [53]. Interestingly, within this Thai isolate, *P28* was expressed at a much higher level than *P25*, when traditionally the opposite has been shown in *P*. *falciparum* and *P*. *vivax* [19]. This is possibly an effect of a gene duplication event documented in *P*. *ovale* [13,54], which has yet to be characterized beyond the genomic level. The expression data outlined here suggest the p28 duplication could be transcriptionally relevant, and have the potential to result in a bona fide phenotypic difference, such as an increased tendency toward sexual development. Importantly, life cycle stages within our sample may not completely reflect the composition of a typical field isolate as parasites underwent leukocyte depletion and short-term culture, both of which can affect parasite stages.

*P*. *ovale* species have hyperexpansion of gene families compared to other primate malaria species, and therefore larger genomes and a higher proportion of their genomes composed of variant antigens [12,55]. There are several multigene families that deserve close examination. *PIR* and *STP1* gene families were strongly under-represented, lacking expression for 23.6% and 65.0% of predicted gene family members, respectively. Conversely, we were able to account for all or nearly all genes for *tryptophan-rich antigen*, *ETRAMP* and *PHIST* gene families (Fig 1D). The *PIR* gene repertoires can vary within different assemblies [56], and we did see some variation in *PIR* and *STP1* families on the genome level. The genome assembly of this Thai isolate was incomplete, and 1238 *PIR* genes and 28 *STP1* genes were found. Because they are subtelomeric, *PIR* and *STP1* genes are especially likely to be missed in sequencing, as whole genome amplification may miss the chromosomal ends. Indeed, we found decreased coverage toward the ends of each chromosome. Besides, the multigene family sequences are also prone to mis-alignment with the reference genome because of their highly repetitive nature. Since the genome of the current Thai isolate is fragmented and incomplete, the genomic repertoires of these multigene families are very likely to be under-represented. This partially explains that 317 *PIR* genes and 28 *STP1* genes previously annotated were not found either in the transcriptome or in the genome assembly of the Thai parasite isolate. Likewise, the *STP1* gene family has around half the number of genes found at the transcriptional level as at the genomic level, suggesting that not all members of this gene family are expressed in blood stages or at the same time. *Plasmodium* parasites express only partial *PIR* repertoires, which would make a lack of transcription for many members intuitive [14]. This Thai isolate contained mixed stages, which may account for simultaneous expression of a large number of *PIR* members. Little has been studied regarding STP1/Surfin protein inter-family dynamics because *P*. *falciparum* and *P*. *vivax* have smaller repertoires with 10 family members [13], while commonly used rodent models have none [57], limiting what can be done *in vitro*. However, it has been shown that STP1 and their SURFIN counterparts are closely related to VAR and PIR proteins [57–59], and they have similar domains suggestive of interaction with the infected RBC surface [59]. Thus, it is likely they may utilize similarly exclusive expression mechanisms for immune evasion. To our knowledge, this is the first evidence suggesting that only a portion of the *STP1* family members are expressed *in vivo* during *P*. *ovale* blood stage infection. In stark contrast, the tryptophan-rich antigen genes were abundantly expressed.

Alternative splicing is an important way of maximizing protein diversity from genetic material, and has been found to enhance antigenic diversity [60]. Over 2500 isoforms identified represent a different model to that predicted from the reference genome. For many of them such as PocGH01_14030000, PocGH01_0804470, PocGH01_11032600 and PocGH01_11024900, the new splicing events change the transcripts drastically and should result in changes of the encoded proteins. It appears that the PHIST and tryptophan-rich gene families have a tendency to encode multiple transcripts with both having a median of two transcripts per gene vs one within all genes (S2 File). This is likely a contributing factor to the PHIST gene family having more predicted whole ORFs than it has transcriptionally active genes (Fig 1D). It is also possible that the reference genome does not adequately represent the repertoire of *P*. *ovale curtisi* gene families, which would similarly result in a discrepancy between mapped reads and *de novo* assembled transcripts.

UTRs play an important role in post-transcriptional processing, but are almost impossible to predict from genomic data alone. Here, at least one UTR has been identified for over half of *P*. *ovale curtisi* genes, some including multiple isoforms with differing lengths. Whereas the average 5’ UTR for the *P*. *ovale* genes was very similar to that documented for *P*. *vivax* [61] (290 bp vs 295 bp), the 3’ UTR was longer (310 bp vs 203 bp). It is possible that differences in UTR lengths between species may represent a means by which *Plasmodium* parasites regulate their development via translational regulation [16,17]. By documenting expected start, end and exonic variation for each gene, this transcriptome study will guide future in-depth studies of individual genes.

Anti-sense transcription is a well-documented regulatory mechanism in *Plasmodium* parasites [15,62,63]. By taking advantage of both a poly-A selected library, enriched for mRNA, and a non-selected library containing all RNA, including that which may act in a purely regulatory manner, we were able to highlight nuance in the relationship between anti-sense and coding RNA. The present study found that 742 genes had at least 25% of transcripts occurring in an anti-sense manner. The abundance of anti-sense transcripts is likely to interfere with the stability of the sense mRNAs and the translational efficiency of the proteins. Consistent with this rationale, we present evidence that there is a correlation between higher levels of anti-sense transcription and lower transcriptional activity within genes with anti-sense transcription, especially at the highest levels of anti-sense activity. Interestingly, the *PIR* gene family members demonstrated a dichotomy pattern of anti-sense activity, significantly enriched in both the “no anti-sense” group, and the group for which over 75% of its transcripts was anti-sense. This is consistent with existing literature suggesting natural anti-sense activity as a mechanism for mutually exclusively expression [15]. Another point of interest was that the correlation between anti-sense reads and expression only has a notable negative correlation when we remove genes with no anti-sense transcripts. Further examination revealed significantly high expression in genes with some, but very little (< 25%), anti-sense transcripts. Future studies are warranted to determine whether anti-sense transcripts in these genes are an important post-transcriptional mechanism of gene regulation.

*P*. *ovale* is understudied despite having distinct biology compared to other malaria species. Here we have presented the first attempt to characterize the transcriptome of a *P*. *ovale curtisi* field sample. The data has supported a vast majority of predicted genes, and remained consistent with known transcriptional regulation in *Plasmodium*, but has also provided evidence of variation from supposed models. Variations could influence our perspective on species-specific models for gene expression. Further, evidence of anti-sense regulation opens the door for future studies into the nature of post-transcriptional regulation within *P*. *ovale curtisi*, and across *Plasmodium* species. In the future, this work could be strengthened by confirmation by similar study in additional *P*. *ovale curtisi* samples. The holistic nature of this work provides a valuable resource to further our understanding of this malaria parasite.

## Supporting information

S1 Fig Thai *Plasmodium ovale* sample is monoclonal *Plasmodium ovale curtisi*. A) Minor allele frequency plot and EstMOI estimate both suggest a very few number of variants where multiple alleles are present. B) Visual inspection of the highly variable MSP1 shows negligible signs of multiple alleles present. C) An assembled MSP1 from the thai field sample has stronger identity to *Plasmodium ovale curtisi* (POC) than *Plasmodium ovale wallikeri* (POW). Percent identity to Thai isolate MSP1 is listed next to each sample, and in the case of *P*. *ovale wallikeri* samples the highest percent identity of two stretches was reported.
(TIF)

S2 Fig Timepoint comparison against *P*. *vivax* orthologs. A total of 160 orthologs for which *P*. *ovale* has only one member were extracted and compared to expression in *P*. *vivax* blood stages. Bluer cells represent overexpression compared to the mixed pool *P*. *vivax* control while red cells represent under-expression.
(TIF)

S3 Fig Relationship between expression level and anti-sense transcription. A) Global correlation between proportion of reads in the anti-sense direction (from the non-selected library) and general expression (from the Poly-A selected library). B) Correlation between anti-sense proportion and expression within genes with any anti-sense transcription. C) TPM within anti-sense divided subgroups. Genes are broken into 5 groups based on the degree of anti-sense transcription: None, Low (0–0.25), Low Mid (0.25–0.5), High-Mid (0.5–0.75), and High (0.75 and higher). Transcription is log transformed TPM. Medians in raw TPM scores are shown for each subgroup. P-values are the product of Wilcoxon rank sum.
(TIF)

S1 Table Alignment of MSP1 from the *Plasmodium ovale* field sample with publicly available MSP1 sequences from *Plasmodium ovale wallikeri* (POW) *and Plasmodium ovale cutisi* (POC). (PDF)

S2 Table Comparison of assembly pipelines. Bolded values represent the best value in each metric. Filtration done by TPM is shown on the right column, while the left column was not filtered. The bottom row shows performance after condensing contigs with 97% similarity, while the top shows performance with no condensation.
(PDF)

S3 Table Cufflinks detected features in Poly-A stranded dataset. (PDF)

S1 File Variation in the *Plasmodium ovale* genome. Variants refer to single nucleotide polymorphisms when compared to the PocGH01 reference. Degree of functional impact (high, moderate or low) is based on potential changes to the amino acid.
(CSV)

S2 File Transcripts master list. Each annotated gene within *P*. *ovale curtisi* is annotated for its abundance in this dataset, the proportion of reads which were in the anti-sense direction, the distinct isoforms found to be notably present, likely UTR locations and relevant GO IDs. Additional tables are subsets of this list are curated groups of genes for sexual stage, RBP and AP2 genes.
(XLSX)

S3 File Functional characterization of genes present and their abundance. Files include gene set enrichment for all GO terms and GO slim terms. An additional enrichment was performed on the genes which were within the top 5% of abundance. Abundance was characterized for both GO terms as well as Malaria Parasite Metabolic Pathways.
(XLSX)
