# Supplementary figures and images for: A glance of the blood stage transcriptome of a Southeast Asian Plasmodium ovale isolate
Source: PLoS Negl Trop Dis. 2019 Nov 15;13(11):e0007850. doi: 10.1371/journal.pntd.0007850 (PMC6881071; doi:10.1371/journal.pntd.0007850)

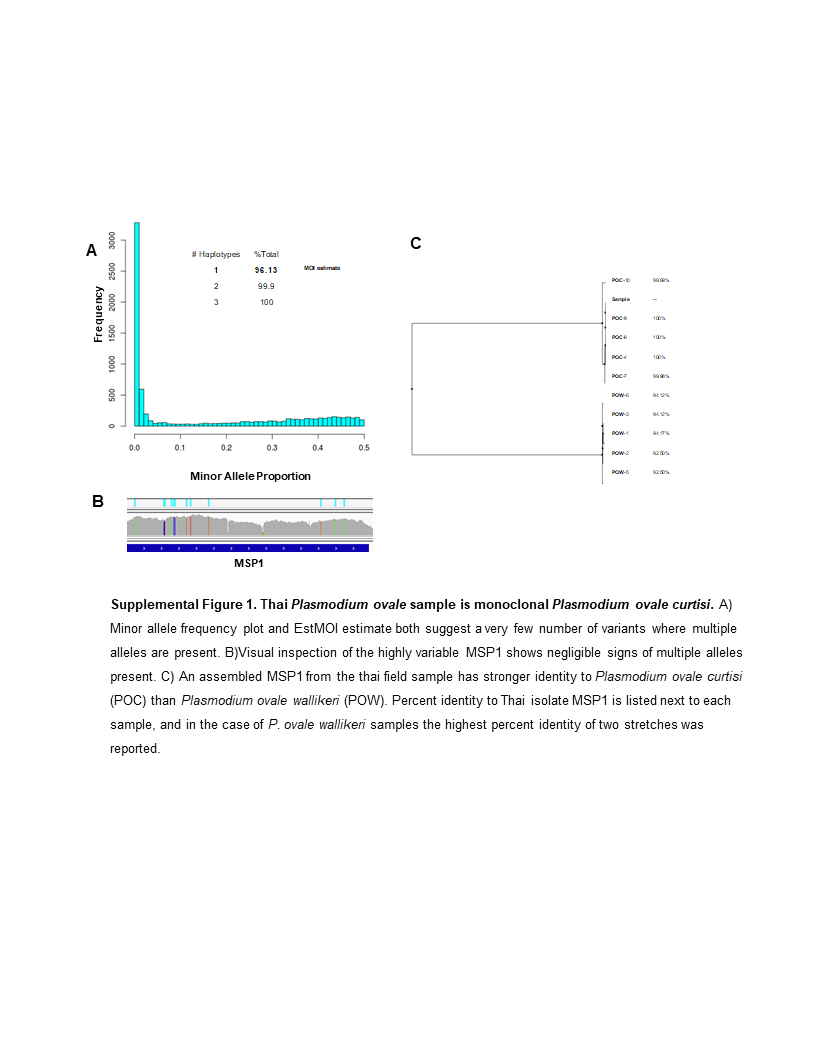

Supplement: S1 Fig — A) Minor allele frequency plot and EstMOI estimate both suggest a very few number of variants where multiple alleles are present. B) Visual inspection of the highly variable MSP1 shows negligible signs of multiple alleles present. C) An assembled MSP1 from the thai field sample has stronger identity to Plasmodium ovale curtisi (POC) than Plasmodium ovale wallikeri (POW). Percent identity to Thai isolate MSP1 is listed next to each sample, and in the case of P. ovale wallikeri samples the highest percent identity of two stretches was reported. (TIF) [file pntd.0007850.s001.tif]

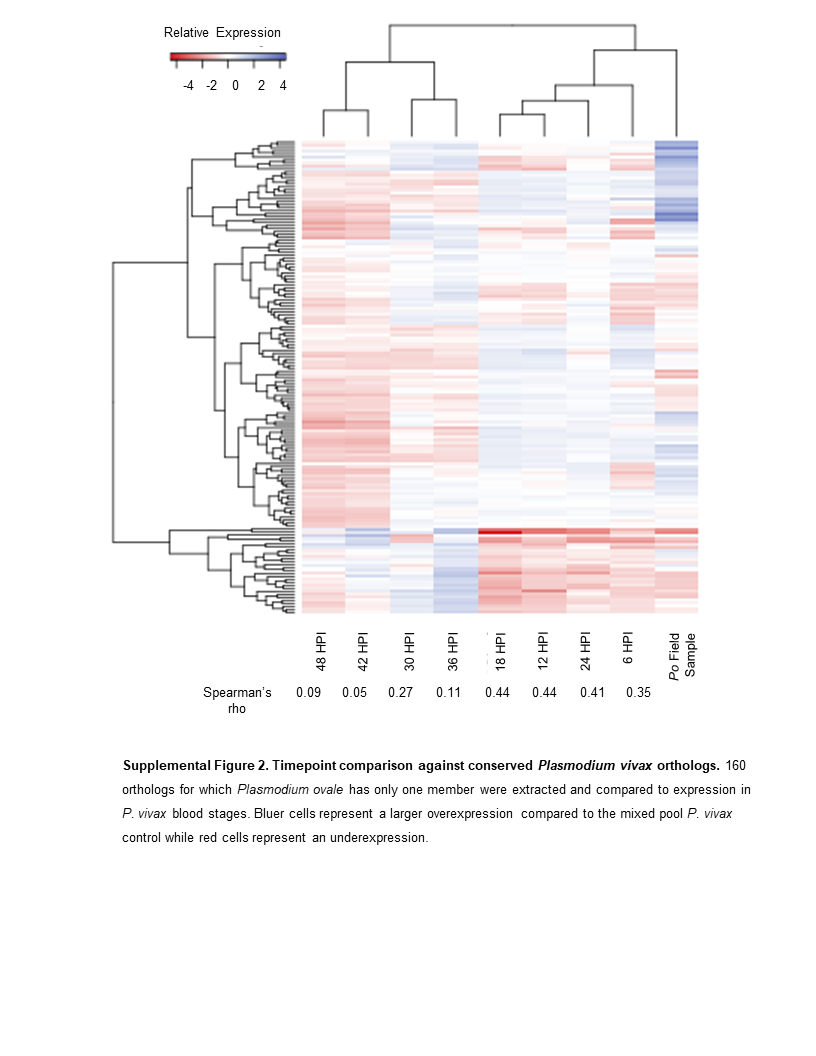

Supplement: S2 Fig — A total of 160 orthologs for which P. ovale has only one member were extracted and compared to expression in P. vivax blood stages. Bluer cells represent overexpression compared to the mixed pool P. vivax control while red cells represent under-expression. (TIF) [file pntd.0007850.s002.tif]

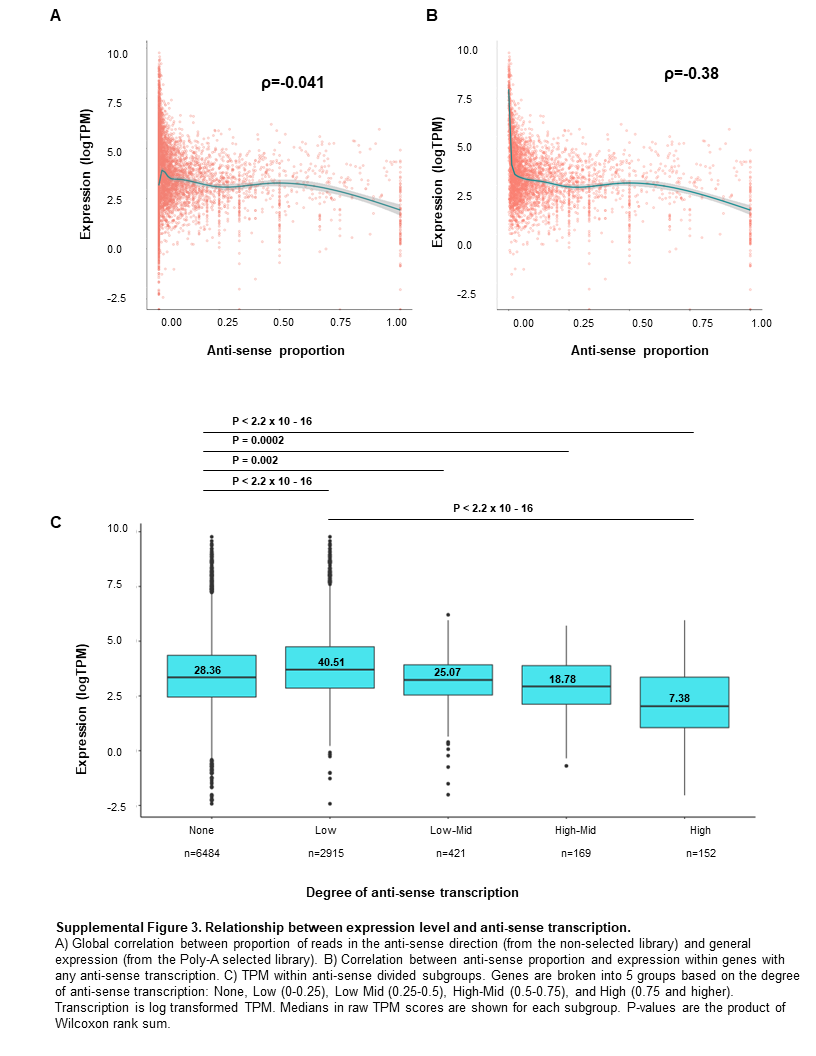

Supplement: S3 Fig — A) Global correlation between proportion of reads in the anti-sense direction (from the non-selected library) and general expression (from the Poly-A selected library). B) Correlation between anti-sense proportion and expression within genes with any anti-sense transcription. C) TPM within anti-sense divided subgroups. Genes are broken into 5 groups based on the degree of anti-sense transcription: None, Low (0–0.25), Low Mid (0.25–0.5), High-Mid (0.5–0.75), and High (0.75 and higher). Transcription is log transformed TPM. Medians in raw TPM scores are shown for each subgroup. P-values are the product of Wilcoxon rank sum. (TIF) [file pntd.0007850.s003.tif]
