# Supplementary material for: A glance of the blood stage transcriptome of a Southeast Asian Plasmodium ovale isolate
Source: PLoS Negl Trop Dis. 2019 Nov 15;13(11):e0007850. doi: 10.1371/journal.pntd.0007850 (PMC6881071; doi:10.1371/journal.pntd.0007850)
Supplement: S1 Table — (PDF) [file pntd.0007850.s004.pdf]

Supplemental Table 1. Alignment results of *Plasmodium ovale* field sample with publicly available *Plasmodium ovale wallikeri (POW) and Plasmodium ovale cutisi (POC)*

Subject	% Identity	Length	Mismatch	Gaps	Qstart	Qend	Sstart	Send	E	Bit
**POC-4**	100	5181	0	0	61	5241	5181	1	0	9568
**POC-8**	100	5181	0	0	61	5241	5181	1	0	9568
**POC-9**	100	5181	0	0	61	5241	5181	1	0	9568
**POC-7**	99.98	5181	1	0	61	5241	5181	1	0	9563
**POC-10**	99.69	5181	16	0	61	5241	5181	1	0	9479
**POW-1**	90.21	2778	240	20	2471	5241	2753	1	0	3594
**POW-1**	94.17	2177	121	4	61	2231	5043	2867	0	3312
**POW-2**	94.12	2177	122	4	61	2231	5016	2840	0	3306
**POW-2**	92	2137	163	4	3112	5241	2136	1	0	2992
**POW-2**	92.5	480	33	2	2471	2950	2726	2250	0	684
**POW-3**	94.12	2177	122	4	61	2231	5016	2840	0	3306
**POW-3**	92	2137	163	4	3112	5241	2136	1	0	2992
**POW-3**	92.5	480	33	2	2471	2950	2726	2250	0	684
**POW-5**	94.12	2177	122	4	61	2231	5016	2840	0	3306
**POW-5**	91.95	2137	164	4	3112	5241	2136	1	0	2987
**POW-5**	92.5	480	33	2	2471	2950	2726	2250	0	684
**POW-6**	94.12	2177	122	4	61	2231	5016	2840	0	3306
**POW-6**	91.95	2137	164	4	3112	5241	2136	1	0	2987
**POW-6**	92.5	480	33	2	2471	2950	2726	2250	0	684
