# Supplementary material for: A glance of the blood stage transcriptome of a Southeast Asian Plasmodium ovale isolate
Source: PLoS Negl Trop Dis. 2019 Nov 15;13(11):e0007850. doi: 10.1371/journal.pntd.0007850 (PMC6881071; doi:10.1371/journal.pntd.0007850)
Supplement: S2 Table — Bolded values represent the best value in each metric. Filtration done by TPM is shown on the right column, while the left column was not filtered. The bottom row shows performance after condensing contigs with 97% similarity, while the top shows performance with no condensation. (PDF) [file pntd.0007850.s005.pdf]

Supplemental Table 2. Comparison of assembly pipelines. Bolded values represent the best value in each metric. Filtration done by TPM is shown on the right column, while the left column was not filtered. The bottom row shows performance after condensing contigs with 97% similarity, while the top shows performance with no condensation.

	Raw	Filtered TPM1+	
Raw	-516285556.2	-514047576.2	RSEM Score
	1203	1181	N50 (All Isoforms)
	1007	1004	N50 (Longest per Gene)
	41192	39921	Contigs
	1082	757	Unfounded Contigs
Condensed (0.97)	-517754189.3	-516153449.5	RSEM Score
	1195	1178	N50 (All Isoforms)
	1050	1048	N50 (Longest per Gene)
	36821	35916	Contigs
	414	188	Unfounded Contigs
