# Supplementary material for: A glance of the blood stage transcriptome of a Southeast Asian Plasmodium ovale isolate
Source: PLoS Negl Trop Dis. 2019 Nov 15;13(11):e0007850. doi: 10.1371/journal.pntd.0007850 (PMC6881071; doi:10.1371/journal.pntd.0007850)
Supplement: S3 Table — (PDF) [file pntd.0007850.s006.pdf]

Supplemental Table 3. Cufflinks detected features

	Cufflinks Class Code	Poly-A Selected Transcripts	Non-selected Transcripts
**Intron Chain Match**	"=”	7350	7397
**Contained**	c	1	0
**Exon Transfrag**	e	257	397
**Intron Transfrag**	I	44	92
**Novel Isoform**	j	2692	2018
**Exonic Overlap**	o	1158	670
**Run-on**	p	461	366
**Antisense Intron Transfrag**	s	26	1
**Intergenic, unknown**	u	2028	1068
**Antisense Exon Overlap**	x	603	258
